# A Tool for Assessment of Animal Health Laboratory Safety and Biosecurity: The Safety Module of the Food and Agriculture Organization’s Laboratory Mapping Tool

**DOI:** 10.3390/tropicalmed3010033

**Published:** 2018-03-14

**Authors:** Beatrice Mouillé, Gwenaelle Dauphin, Lidewij Wiersma, Stuart D. Blacksell, Filip Claes, Wantanee Kalpravidh, Youssouf Kabore, Sharon Hietala

**Affiliations:** 1 Food and Agriculture Organization of the United Nations, Rome 00153, Italy; Lidewij.Wiersma@fao.org; 2 CEVA Animal Health, Libourne 33500, France; Gwenaelle.Dauphin@ceva.com; 3 Center for Tropical Medicine and Global Health, University of Oxford, Oxford OX3 7FZ, UK; Stuart@tropmedres.ac; 4 Mahidol-Oxford Tropical Medicine Research Unit, Faculty of Tropical Medicine, Mahidol University, Bangkok 10400, Thailand; 5 Food and Agriculture Organization of the United Nations, Regional Office for Asia and the Pacific, Emergency Center of the Transboundary Animal Diseases, Bangkok 10200, Thailand; filip.claes@fao.org (F.C.); wantanee.kalpravidh@fao.org (W.K.); 6 Food and Agriculture Organization of the United Nations, Dakar 999066, Senegal; Youssouf.Kabore@fao.org; 7 Department of Medicine and Epidemiology, School of Veterinary Medicine, University of California, Davis, CA 95616, USA; skhietala@ucdavis.edu

**Keywords:** biosafety, biosecurity, animal health, laboratory assessment

## Abstract

The Laboratory Management Tool (LMT) is a standardized spreadsheet-based assessment tool developed to help support national, regional, and global efforts to maintain an effective network of animal health and veterinary public health laboratories. The safety and biosecurity module of the LMT (LMT-S) includes 98 measures covering administrative, operational, engineering, and personal protective equipment practices used to provide laboratory safety and biosecurity. Performance aspects of laboratory infrastructure and technical compliance considered fundamental for ensuring that a laboratory is able to appropriately function in a safe and biosecure manner are systematically queried and scored for compliance on a four-point scale providing for a semi-quantitative assessment. Data collected is used to generate graphs and tables mapping levels of compliance with international standards and good practices, as well as for documenting progress over time. The LMT-S was employed by trained auditors in 34 laboratories located in 19 countries between 2015 and 2017. The tool is intended to help standardize animal health laboratory assessments, document compliance with recognized laboratory safety and biosecurity measures, serve as a self-help and training tool, and assist global laboratory development efforts by providing an accurate measurement of laboratory safety and biosecurity at local, national, and regional levels.

## 1. Introduction

Veterinary laboratories are an important component of global animal health, as well as public health, through their involvement in disease surveillance, diagnosis, and control. These laboratories play roles in detection of zoonotic and animal-specific diseases, in food safety, and in production and development of vaccines and therapeutic strategies for both humans and animals. By the nature of the work they perform, veterinary laboratories routinely work with materials that, if inappropriately handled or improperly contained, can pose a range of health and economic threats. In addition to chemical safety and radiation safety, laboratories globally must be critically aware of biosafety, defined as ‘the principles and practices for prevention of unintentional exposure to or release of biological materials’ [1], and biosecurity, defined as ‘the control of biological materials within laboratories that prevent their loss, theft, misuse, unauthorized access, or intentional release’ [1]. Complete and well-functioning laboratory biosafety and biosecurity programmes are critical not only to protect laboratory workers from inadvertent exposures and potential infection, but importantly, to protect the local and regional animal populations, the public, and the environment from accidental or intentional release and spread of biological hazards handled by laboratories.

The Food and Agriculture Organization of the United Nations (FAO) is committed to building strong and effective veterinary care, disease diagnosis, and disease control to support the food supply infrastructure internationally, in part through assisting veterinary laboratories and their national partners in identifying gaps in veterinary laboratory functionality and in supporting laboratory capacity-building on a global level. In support of this goal, in 2010 the FAO developed a laboratory assessment approach targeting veterinary laboratories. The approach, named the Laboratory Mapping Tool (LMT), promoted the use of standardized laboratory assessments in order to identify and monitor gaps in laboratory capacity and functionality, and ultimately to assist decision-makers in better defining the targets and mechanisms for both local and global laboratory capability and capacity-building.

The FAO LMT is based on a questionnaire that generates a laboratory profile or ‘map’ by scoring performance on the aspects of laboratory management, infrastructure, and technical performance that are considered to be fundamental for ensuring that a laboratory is able to appropriately handle samples, to detect and report on animal diseases, and ultimately to facilitate a rapid response to disease threats. The LMT approach is designed to have the flexibility to document laboratory functionality and capacity at local, national, and regional levels; and to be used by either external assessors or by laboratories in their own self-assessments.

The LMT design concept was to develop a series of LMT modules that would address critical management and facility requirements, such as quality system management, specific disease testing capacities, laboratory safety, biosafety, and biosecurity, among others. The first of the LMT modules developed and released was the LMT-Core, which was designed to identify strengths and gaps in five broad categories: a general laboratory profile; infrastructure, equipment and supplies; technical performance; quality assurance, biosafety and biosecurity; and collaborations and networking. Twenty-four individual countries, having one or more laboratories each, agreed to participate in vetting of the LMT-Core. In 2014, the tool was made publicly available through the FAO web page [2]. A segment of the LMT-Core specifically focuses on laboratory safety and security practices, with an emphasis on biosafety and biosecurity within the overall management and infrastructure of a laboratory. Of the 108 sub-categories (topics) included in the LMT-Core questionnaire, twelve percent (13/108) address infrastructure that supports laboratory safety and security practices in general, and an additional 24% (26/108) relate to biorisk assessment, biosafety, and biosecurity-specific practices that have been recommended in manuals and guidance materials produced by the World Health Organization [3,4], U.S. Centers for Disease Control [5], World Organisation for Animal Health (OIE) [1], and the European Committee for Standardization [6,7], among others.

Despite the recent growth in disease detection capacity globally, the design and implementation of associated laboratory safety and biosecurity programmes lags and is inconsistent globally due to a variety of factors that include differences in national and local infrastructures, available funding and priorities, regulatory frameworks, and accessibility to expertise, training and equipment resources [8]. Additionally, documented failures of established laboratory biosafety systems [9], including the veterinary-specific 2007 outbreak of foot and mouth disease in the United Kingdom [10], emphasize the critical role for monitoring and assessment [11,12] as components of biosafety and biosecurity programme and practice management. The FAO LMT-Core assessments performed to date have identified recurrent weaknesses in the implementation or maintenance of safety and biosecurity practices, particularly in some of the more resource-limited countries. Those findings support the need for a standardized laboratory assessment tool that can serve an important role, not only in documenting the level of safety and biosecurity compliance for individual laboratories, but also in ascertaining the level of need on a local, national, and regional basis in support of ongoing efforts for global disease detection and response capacity-building. The goal of the LMT-Safety Module (LMT-S) presented here was to develop a standardized laboratory assessment tool for a more detailed evaluation of the current and ongoing status of laboratory safety, including chemical and radiation safety, and with a strong focus on biosafety and biosecurity in veterinary laboratories.

## 2. Materials and Methods

The LMT-S tool is formatted as a questionnaire embedded in a spreadsheet (Microsoft Excel 2007). Responses entered by assessors are automatically converted into an overall safety and biosecurity score. The calculated score is accompanied by tabulated summaries and graphic depictions of the laboratory’s safety and biosecurity strengths and weaknesses. The LMT-S, which was modeled after the FAO LMT-Core module, has questions which specifically target laboratory safety, biosafety, and biosecurity in the context of administrative activities, operational procedures, engineering, and personal protective equipment (PPE) used to control accidental or intentional release of biological materials and to respond to potential adverse events in the laboratory. The LMT-S was designed to be used either by trained external assessors during on-site assessments used for establishing baseline laboratory safety and biosecurity performance and for measuring progress of post-intervention activities, or for use by laboratories in self-assessment, monitoring, and continuous improvement of their own safety and biosecurity-related practices. Similar to the LMT-Core, the LMT-S is available in spreadsheet format and has also been piloted in Web-based and mobile applications available in English, French, and Thai, with translation to other languages provided upon request. The LMT-S questionnaire is formatted into 4 safety and biosecurity areas containing 20 related categories and 98 subcategories which define specific management activities, laboratory practices, facility and equipment resources, and personal protective equipment (PPE) use (Table 1: Areas and Categories covered by the LMT-S).

The terminology for the 4 LMT-S areas and 20 categories is consistent with that found in the World Organisation for Animal Health (OIE) chapter ‘Biosafety and Biosecurity: Standard for managing biological risk in the veterinary laboratory and animal facilities’ [1]. Each of the 98 subcategories addresses a specific laboratory practice that is relevant to the individual category. Briefly, the administrative area queries safety and biosecurity control measures involving management-level programmes and policies relating to human resources, procedures, record-keeping, assessment, and reporting as needed to ensure a safe and biosecure work environment. Operations addresses the safety and biosecurity control measures that ensure day-to-day activities and actions are consistent with established policies and objectives. The engineering area focuses on the physical features of the laboratory designed to remove, or to place a barrier to, biological or physical hazards. The PPE area specifically queries the availability and use of the clothing and equipment designed to protect the worker’s body.

Each of the 98 subcategories have four scoring options, with the highest level of compliance and activity receiving a score of 4 and the most basic level of activity or awareness receiving a score of 1. Each scoring option is described in the form of safety and biosecurity practices or procedures (Table 2: Example of scoring options for the LMT-S subcategory question ‘Disposable glove usage’). The scoring option of “Not applicable” is additionally provided, in order to remove any subcategory topics that may not apply to a specific laboratory from the calculation of a laboratory’s overall safety score, e.g., biosafety level (BSL)-3 containment practices that would not apply in a BSL-2 facility. To help standardize the assessment process and assist assessor(s) with scoring, written guidance is provided within the tool for each subcategory. These guidance comments are included adjacent to each subcategory and direct the assessor to specific documents, records, practices, procedures, facility engineering, and PPE use that the assessor should review or observe as objective evidence for the subcategory scoring. Space is additionally provided within the spreadsheet to capture narrative input(s), as needed, to document or further explain assessment observations. In order to allow for comparison over time or to compare different assessors’ findings (e.g., as a training tool), the LMT-S spreadsheet allows scoring from 3 different assessments and automatically provides both summary and graphic results comparing the multiple assessments.

The LMT-S file includes 6 spreadsheet tabs: (1) the Safety Index tab briefly describes the LMT-S tool; (2) a guideline for users; (3) the Laboratory Information tab used to capture information regarding the laboratory being assessed; (4) the Safety Module tab containing the questionnaire and capturing the assessment data; and (5) the Safety Summary tab containing the tabulated and graphically-depicted information automatically generated from the results that were entered for the assessment(s). The sixth tab provides FAO copyright and associated information related to development and rights to access the LMT-S module.

**Figure 1:**
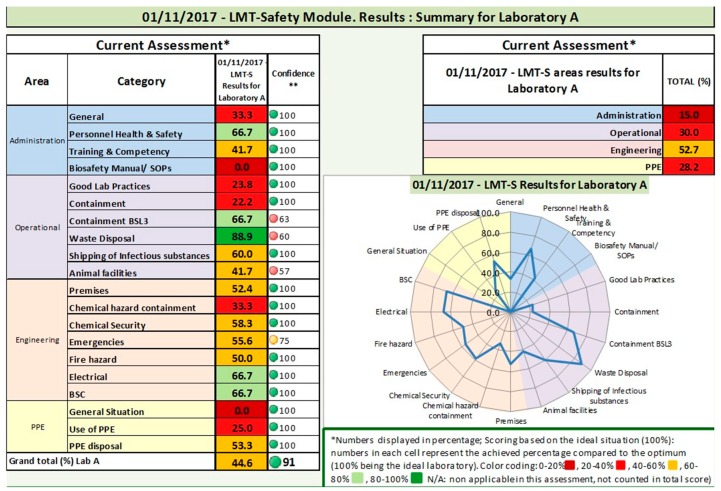
Example of results of an LMT-S assessment conducted in region B.

Scoring within the LMT-S is based on a percentage of an optimum score of 100%, which represents an ideal situation, i.e., a laboratory that would score 4 for all the sub-categories assessed. A confidence score is additionally calculated from the percentage of subcategory questions that are not completed (i.e., left blank) by the assessor. Completion of 0–69% of the questionnaire provides a low confidence score, 70–89% a medium confidence score, and 90–100% completion of the questionnaire is ranked as reliable. The confidence score is reported as a component of the summary results. The laboratory safety and biosecurity data is tabulated and also graphically depicted using a color-coded radar-style chart, with rankings allocated to 0–20%, 20–40%, 40–60%, 60–80% and 80–100% as compared to the optimum 100% benchmark score for each area and category (Figure 1). When multiple assessments are provided (e.g., over time, by different assessors, before and after intervention activities), the results are tabulated by assessment, by area and by category, and additionally presented graphically by area and by category as a compilation allowing for comparison of up to three assessments.

Training on use of the LMT-S followed by laboratory assessment missions were conducted in two geographically distinct global regions, with a total of 19 countries and 34 laboratories participating. Assessor training in a third geographic region was scheduled, but not completed for inclusion in the current data analysis. The laboratories evaluated included the range of facilities, including well-funded and well-resourced regional and national laboratories, as well as laboratories that would be classified as resource-limited. The identities of the laboratories are accessible to the FAO administration and to participating laboratories, but are not reported here to protect the confidentiality of the assessment process.

## 3. Results

Of the 34 laboratories participating in the pilot use of the LMT-S, 17 were located in geopolitical region A and 17 in region B. A total of 19 (region A = 4, region B = 15) countries participated in the evaluation and use of the LMT-S during calendar years 2015 through 2017. Follow-up or post-intervention assessments were not completed for all laboratories, so only initial assessment findings are analyzed and reported here.

Results from the initial LMT-S assessments indicated that safety and biosecurity procedures and practices were minimally adequate for the risk of procedure(s) being performed, and overall below the performance recommended by internationally-recognized safety and biosecurity guidance documents, including those identified in the Global Health Security Agenda Prevent project (Action Package Prevent 3) launched in 2014, involving approximately 50 partner countries plus contributing international organizations FAO, IAEA, Interpol, OIE, and WHO [13].

The average score for region A laboratories was 41.3% (±14.2, range 13–64%) and for region B was 28.1% (±17.7, range 3–74%) (Table 3: Summary Scores from initial LMT-S assessments). Overall, administrative activities (i.e., safety and biosecurity controls), which are defined by laboratory policies and high level laboratory management (e.g., designation of safety and security officers, personnel health and safety programs, training and competency programs, etc.), and appropriate engineering controls (e.g., containment of fire, chemical, and electrical hazards; facility design; biosafety cabinet placement and certifications; etc.), were the predominant areas of weakness in both regions. The strongest relative scores for both regions were in the area involving PPE; however, even as the strongest scoring area the LMT-S findings indicated PPE availability, use, and disposal practices were not optimal for the laboratories in the two regions participating in the pilot study.

In region A, the lowest scoring safety and biosecurity measures were associated with chemical hazard containment and security (average laboratory score 14% and 28% respectively); electrical hazard control (34%); general administrative oversight of potentially high risk agents and toxins (36%); and access to current and language-appropriate biosafety manuals and standard operating procedures (SOPs) in the facility (36%). The subcategories of greatest strength included placement, certification, and correct use of biosafety cabinets (average laboratory score 73%); all other measures fell below a score of 60%, indicating the need for enhanced focus on laboratory safety and biosecurity. The largest discrepancies between individual laboratories in Region A included handling of electrical hazards, general PPE training and use, and maintenance of training and competency practices.

In region B, among the lowest scoring measures were emergency preparedness (average laboratory score 8%); chemical hazard security (12%); fire hazard control (15%); and availability of current language-appropriate biosafety manuals and SOPs in the laboratory facility (16%). Categories and subcategories of greatest strengths for laboratories in region B received scores that would still rank them in need of significant improvement, and included appropriate PPE disposal practices (50%), shipping of infectious materials (45%); and appropriate PPE use (41%). The largest discrepancies between individual laboratories in Region B were in PPE use and availability; biological safety cabinet (BSC) use; waste disposal practices; and the maintenance of training and competency practices. National and reference laboratories routinely scored higher overall in both regions, presumably due to increased access to funds and related resources.

**Figure 2:**
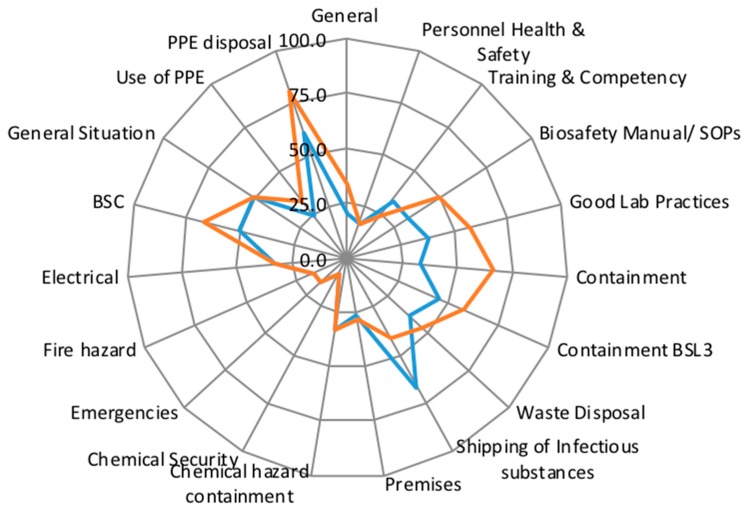
Radar graph comparing an initial (blue line) and post-intervention assessment (red line).

Because not all participating laboratories had completed a second follow-up assessment after initial use of the LMT-S tool, comparative data is not presented here. However, for the laboratory management that did complete follow-up assessments with the goal of gauging progress and effectiveness of interventions, users noted overall that scores improved following implementation of the LMT-S due to increased awareness of safety and biosecurity issues, and from identification of obtainable measures as identified in the LMT-S that would increase compliance and advance the laboratory to the next higher scoring category (e.g., enforcement of GLP practices, systematic management of hazard containment and waste disposal). Figure 2 provides an example radar graph comparing baseline (initial assessment) and post-intervention scores from a region B laboratory that completed a follow-up assessment. The General Laboratory Safety and Security score of the laboratory increased from 43.8 to 55.6, largely reflecting significant improvement of compliance in operational practices.

## 4. Discussion

There are a variety of internationally-recognized assessment tools and compliance checklists available to veterinary laboratories, each designed to fulfill a specific purpose and all ultimately targeting strengthening infrastructure that will help to better serve public and veterinary health, as well as animal-related economies. In addition to the LMT-Core, the World Health Organization and the Centers for Disease Control provide compliance checklists and recommendations for assessing laboratory competencies, including biosafety practices [3,5]. The LMT-S tool is unique in specifically targeting the areas of safety and biosecurity in veterinary laboratories as a critical component of the veterinary and veterinary public health infrastructure globally. The LMT-S is designed to be used by both external assessors, and by internal laboratory management for self-assessment and training (awareness) purposes.

When used by external assessors, the LMT-S provides standardized and objective data that can be used by national and international authorities for raising awareness concerning capacity and limitations in a laboratory or network of laboratories, and ultimately for developing the priorities and plans aimed at strengthening the veterinary laboratory network overall. As an assessment tool, the LMT-S can be paired with the broader-focused FAO LMT-Core tool to identify and more precisely define opportunities for safety and biosecurity improvements in the infrastructure, management, and operations of a specific laboratory or laboratory network. Since the safety and biosecurity practices evaluated by the LMT-S are fully consistent with the OIE standard on a risk-based approach to biosafety and biosecurity, the tool also coordinates well and provides specific detail supporting the OIE Tool for Evaluation of Performance of Veterinary Services (OIE PVS Tool), which is used by national veterinary services authorities to assess their overall national veterinary infrastructure and administration. In a similar vein of providing focused and valuable detailed assessment of veterinary laboratory safety and biosecurity, the LMT-S can provide standardized data necessary for informing the Global Health Security Agenda Joint External Evaluation (GHSA JEE). The GHSA JEE is a well-recognized comprehensive assessment with a One Health focus that is performed at a national level, and used by countries to identify the strengths and weaknesses within their health system (i.e., veterinary, public, environmental) in order to prioritize and access opportunities, including funding for capacity development in disease prevention, detection, and response.

With regional biosafety programmes currently implemented in both region A and B, the results of the standardized LMT-S assessments can be used to document safety and biosecurity compliance, and can be shared between countries as they participate in national and regional discussions on priorities for capacity development, resource sharing, and intervention strategies.

The LMT-S assessment pilot conducted in 19 countries in two geopolitical regions affirmed that engineering controls, including construction, certification, and maintenance of high-level containment facilities is an ongoing challenge, particularly in resource-restricted countries. Importantly, the findings also pointed to key opportunities for relatively accessible activities that can be used to improve safety and biosecurity practices in these laboratories. Examples of non-resource-intensive opportunities identified by the initial round of LMT-S data include provision of task-specific training and information that addresses the essential competencies needed by laboratory personnel in order to work safely with biologic materials and other hazards that are routinely found in veterinary laboratories (e.g., access to language-appropriate biosafety information, PPE don–doff training, waste labeling and disposal practices, chemical and biologic agent inventory practices, emergency and informational signage, chemical and infectious agent storage procedures, etc.).

In addition to the guidance notes included with each subcategory, training sessions held prior to use of the LMT-S provided information not only for how to use the tool, but generated a shared understanding of the terminology and auditing expectations in order to standardize the assessment process and ranking options for the various safety and biosecurity elements. Local assessors participated, either formally or as observers, in the individual laboratory assessments, and in the limited circumstances where both external and internal assessors simultaneously scored laboratories, the rankings were consistent. In addition to user training sessions, the ability to compare separate assessments is available within the LMT-S, and should continue to serve as an informal training tool allowing new assessors to compare their overall and individual element scoring to more experienced assessors. It is noted that ongoing efforts to maintain consistency and standardization in scoring will be critical to the successful long-term use of the LMT-S.

When used directly by a laboratory, in addition to self-assessment, the LMT-S provides a means of self-help for safety and biosecurity training, evaluation, and goal-setting. The LMT-S tool is formatted into safety and biosecurity areas, categories, and subcategories that define specific laboratory programmes and practices, and so beyond use as an assessment tool, the LMT-S is an easily-accessible educational and safety programme awareness-tool. The four scoring options provided for each subcategory supply a progression with each increasing score identifying the additional practice or level of implementation and management required to advance to a higher score, and therefore can have a role in laboratory training, including self-training, to identify specific steps or opportunities to move toward a more optimal safety and biosecurity programme. The LMT-S guidance notes provided with each subcategory identify appropriate documentation required in order to demonstrate compliance with a recommended practice, and although directed to the assessment process, these notes can also be used by laboratories needing instruction on appropriate documentation, including schedules and certifications, that are critical to a well-functioning safety and biosecurity programme.

Laboratory management using the LMT-S noted the value in being able to self-evaluate and learn from the tool the measures needed to enhance their level of safety and biosecurity compliance, as well as to measure progress and effectiveness of intervention strategies. Specific user-based requests for expanding use of the LMT included requests for a basic module to assist laboratories in implementing an initial safety and security programme, modification of the current LMT to allow comparison of more than three assessments, and access to a restricted-access LMT Web-portal that would allow sharing, at the country’s discretion, with the FAO administration and with partner countries. In response, FAO is currently refining and testing a Web portal that allows countries to compile and archive all assessments for each individual laboratory in their national laboratory network; provides automated statistical analysis of the data captured; and allows countries to compare their LMT status (scores) on an anonymous basis at national, regional, and global levels. A goal of the LMT Web-portal is to provide laboratory’s further incentive for improving laboratory functionality and capacity, and ultimately help laboratories enhance their profile nationally, regionally, and globally.

The LMT-S was specifically developed with the goal of ascertaining and improving the level of laboratory safety and biosecurity in support of global disease detection and response capacity-building. The LMT-S has been demonstrated during pilot testing to be an accessible multipurpose tool for documenting and improving laboratory safety and biosecurity. The tool fills an important niche in helping to provide a safe and biosecure laboratory environment through standardized assessment and documentation, which in turn provide critical awareness and objective data useful in national and international health, including laboratory funding and prioritization activities. The LMT-S has the added benefit of generating safety and biosecurity awareness and providing related training opportunities outside of the formal assessment process.

## Figures and Tables

**Table 1:** Areas and categories covered by the safety and biosecurity module of the Laboratory Management Tool (LMT-S Module).

Area	Category	Number of Associated Subcategories (Total 98)
Administration	General	5
	Personnel health and safety	4
	Training and competency	4
	Biosafety manual/Standard operating procedures (SOPs)	2
Operations	Good lab practices	7
	Containment	6
	Containment BSL3	8
	Waste disposal	5
	Shipping of infectious substances	5
	Animal facilities	7
Engineering	Premises	7
	Chemical hazard containment	6
	Chemical security	4
	Emergencies	4
	Fire hazard	4
	Electrical	4
	Biological safety cabinet (BSC)	3
Personal protective equipment (PPE)	General situation	4
	Use of PPE	4
	PPE disposal	5

**Table 2:** Example of scoring options for the LMT-S subcategory question ‘Disposable glove usage’.

Score	Laboratory Practice
Score: 4	Disposable gloves (and double gloves when appropriate) are worn per chemical/pathogenic agent-specific or procedural SOP, are inspected frequently for contamination or loss of integrity, and are not reused.
Score: 3	Disposable gloves are worn whenever working with potentially toxic or infectious materials and biologicals, are changed frequently during a work shift, and are not reused.
Score: 2	Gloves are required whenever handling potentially toxic/infectious materials and biologicals. Disposable gloves may be worn for all or most of a work shift and are not reused.
Score: 1	Gloves are generally worn when working with toxic/infectious materials; disposable gloves may be washed and reused.
Additional information for the assessor	Documentation that can be checked during the assessment may include training materials, laboratory-specific or general biosafety manuals or SOPs, don-doff procedures; on-site observation.

**Table 3:** Summary scores from initial LMT-S.

Safety and Biosecurity Category	Region A (*n* = 17 Laboratories)	Region B (*n* = 17 Laboratories)
Overall Lab Safety and Biosecurity	41.3 (±14.2); range 13–64%	28.1 (±17.7); range 3–77%
Administrative Controls	38.1 (±2.6); range 18–56%	21.1 (±16.1); range 0–56%
Operational Controls	48.1 (±7.8); range 19–71%	32.7 (±18.9); range 16–87%
Engineering Controls	35.2 (±18.6); range 6–60%	20.9 (±16.9); range 0–68%
PPE	50.6 (±7.0); range 17–75%	42.4 (±25.7); range 6–77%
